# Analysis of factors related to osteoporotic vertebral fracture in prostate cancer patients

**DOI:** 10.1007/s12672-024-00886-5

**Published:** 2024-02-13

**Authors:** Shunfa Huang, Lilan Wu, Shuting Lin, Siqing Cai, Jianjun Zhou

**Affiliations:** 1 Department of Radiology, Zhongshan Hospital (Xiamen), Fudan University, No.668 Jinhu Road, Huli District, Xiamen, 361015 China; 2 Department of Radiology, The Second Affiliated Hospital of Fujian Medical University, No.34 Zhongshan North Road, Licheng District, Quanzhou, 362000 China; 3 Xiamen Municipal Clinical Research Center for Medical Imaging, No.668 Jinhu Road, Huli District, Xiamen, 361015 China

**Keywords:** Prostate cancer, Osteoporosis, Vertebral fracture, Androgen deprivation therapy

## Abstract

**Objective:**

This study was aimed at exploring the osteoporotic vertebral fracture rate and the related causal factors in prostate cancer patients before and after treatment.

**Methods:**

One hundred prostate cancer patients were recruited in this study. One hundred men without prostate cancer history were selected as the control group. The study was approved by the Medical Ethics Committee under Ethics number B2021-373R and the requirement for the informed consent was waived. The T4-L1 vertebral body of the case group and the control group before and after treatment was evaluated according to Genant’s semi-quantitative method. The difference in vertebral body fracture rate between the case group and the control group and the changes in vertebral body fracture rate before and after treatment among the case group were compared. They were grouped according to age, body mass index (BMI), prostate-specific antigen (PSA) levels, Gleason grade, and androgen deprivation therapy (ADT). Univariate and multivariate logistic regression models were used to determine the factors significantly associated with vertebral fracture rate in prostate cancer patients.

**Results:**

The prevalence of vertebral fracture was 16% and 31% in prostate cancer patients before and after treatment, respectively, and 29% in the control group. The vertebral fracture rate of the patients before treatment significantly differed that of the control group and the patients after treatment. Univariate analysis showed that age, PSA levels, and treatment parameters were the significant influencing factors of vertebral fracture rates. Multivariate logistic regression analysis showed that age was the main influencing factor of vertebral fracture rates.

**Conclusion:**

Osteoporotic vertebral fractures in patients with prostate cancer was associated with many factors. And the incidence of vertebral fracture in prostate cancer patients after ADT was significantly higher than that before treatment.

## Introduction

Osteoporotic fracture is one of the main causes of disability and death in elderly patients. With the increasing social age of the population, the prevalence of osteoporosis has significantly increased. In the past, the diagnostic and treatment modalities for osteoporosis focused more on female patients than male patients. However, men over the age of 50 years have a lifetime risk of osteoporotic fracture of 13%, similar to the lifetime risk of prostate cancer [1]. Since the discovery of the effect of castration (surgery-based or drugs-based) on prostate cancer in 1941, androgen deprivation therapy (ADT) has become one of the cornerstones of prostate cancer treatment. ADT not only reduces the recurrence rate of prostate cancer patients but also prolongs their lives [2]. Due to the improved detection rate and treatment of prostate cancer, more patients are receiving ADT than ever before; however, the course of treatment is longer than ever before [3]. ADT reduces estrogen and testosterone levels by disrupting the hypothalamic-pituitary–gonadal axis. However, its mechanism of action also leads to a range of side effects, such as sexual dysfunction, hot flashes, cognitive decline, insulin resistance, anemia, ischemic heart disease, muscle atrophy, and increased bone resorption [4]. Increased bone resorption leads to decreased bone mineral density (BMD) and bone structural damage, leading to an increased risk of osteoporotic fracture. Previous studies have reported that the reduction of BMD after the first year of ADT is in the range of 2–8% [5]. With subsequent ADT, BMD will still be lost at a rate of 2–4.5% per year. Long-term ADT has significant negative effects on the bone health of prostate cancer patients [6]. The purpose of this study was to explore the changes in osteoporotic vertebral fracture rate and related influencing factors in prostate patients before and after they received ADT. With this information, the patients can be better advised to prevent therapeutic bone loss and reduce the rate of vertebral fractures through adequate lifestyle changes and medication. Thus, our results will be instrumental in improving the quality of life of prostate cancer patients postoperatively.

## Materials and methods

### Study population

A total of 100 prostate cancer patients with complete clinical data from July 2013 to March 2020 were selected as the case group. The study was approved by the Medical Ethics Committee under Ethics number B2021–45 and the requirement for the informed consent was waived. The average age and BMI of the patients were 69.68 years (range: 48–88 years) and 22.94 kg/m^2^ (15.97–33.91 kg/m^2^), respectively. From January 2020 to August 2020, 100 men without a history of prostate cancer were selected as the control group. The average age of the patients was 68.59 years (range: 47–88 years). The age of the two groups was matched. The basic information of the prostate cancer patients recruited in this study is listed in Table 1.

**Table 1:** Basic information about the patients

Basic data (n = 100)	Prostate cancer patients before treatment		Prostate cancer patients after treatment	
	Without vertebral fracture (%)	With vertebral fracture (%)	Without vertebral fracture (%)	With vertebral fracture (%)
Age				
≤ 70	46 (86.8)	7 (13.2)	41 (77.4)	12 (22.6)
> 70	38 (80.9)	9 (19.1)	28 (59.6)	19 (40.4)
PSA (ng/mL)				
≤ 4	2 (100)	0 (0)	0 (0)	2 (100)
(4–10)	15 (68.2)	7 (31.8)	15 (68.2)	7 (31.8)
(10–20)	14 (93.3)	1 (6.7)	14 (93.3)	1 (6.7)
> 20	53 (86.9)	8 (13.1)	40 (65.6)	21 (34.4)
Gleason grade				
≤ 7	41 (77.4)	12 (22.6)	32 (60.4)	21 (39.6)
> 7	43 (91.5)	4 (8.5)	37 (78.7)	10 (21.3)
Treatment time (years)				
≤ 2	58 (80.6)	14 (19.4)	50 (69.4)	22 (30.6)
> 2	26 (92.9)	2 (7.1)	19 (67.9)	9 (32.1)
ADT				
Yes	81 (88.0)	11 (12.0)	66 (71.7)	26 (28.3)
No	3 (37.5)	5 (62.5)	3 (37.5)	5 (62.5)

*PSA* prostate-specific antigen, *ADT* androgen deprivation therapy

### Inclusion criteria

Patients whose diagnosis was confirmed by pathology and immunohistochemistry and patients who had standard lateral chest radiographs before and after treatment. The control group was healthy with no history of cancer.

### Exclusion criteria

(1) Patients taking glucocorticoids, vitamin D, calcium, and other drugs for a long time; (2) Patients with liver and kidney diseases, hyperparathyroidism, immune diseases, etc.; (3) Patients with high-energy trauma, post-traumatic deformity, tuberculosis, Scheuermann’s disease, congenital spinal deformity, degenerative scoliosis or unclear lateral chest X-ray film, and tumor metastasis before and after treatment [7].

### Research method

**Fig. 1:**
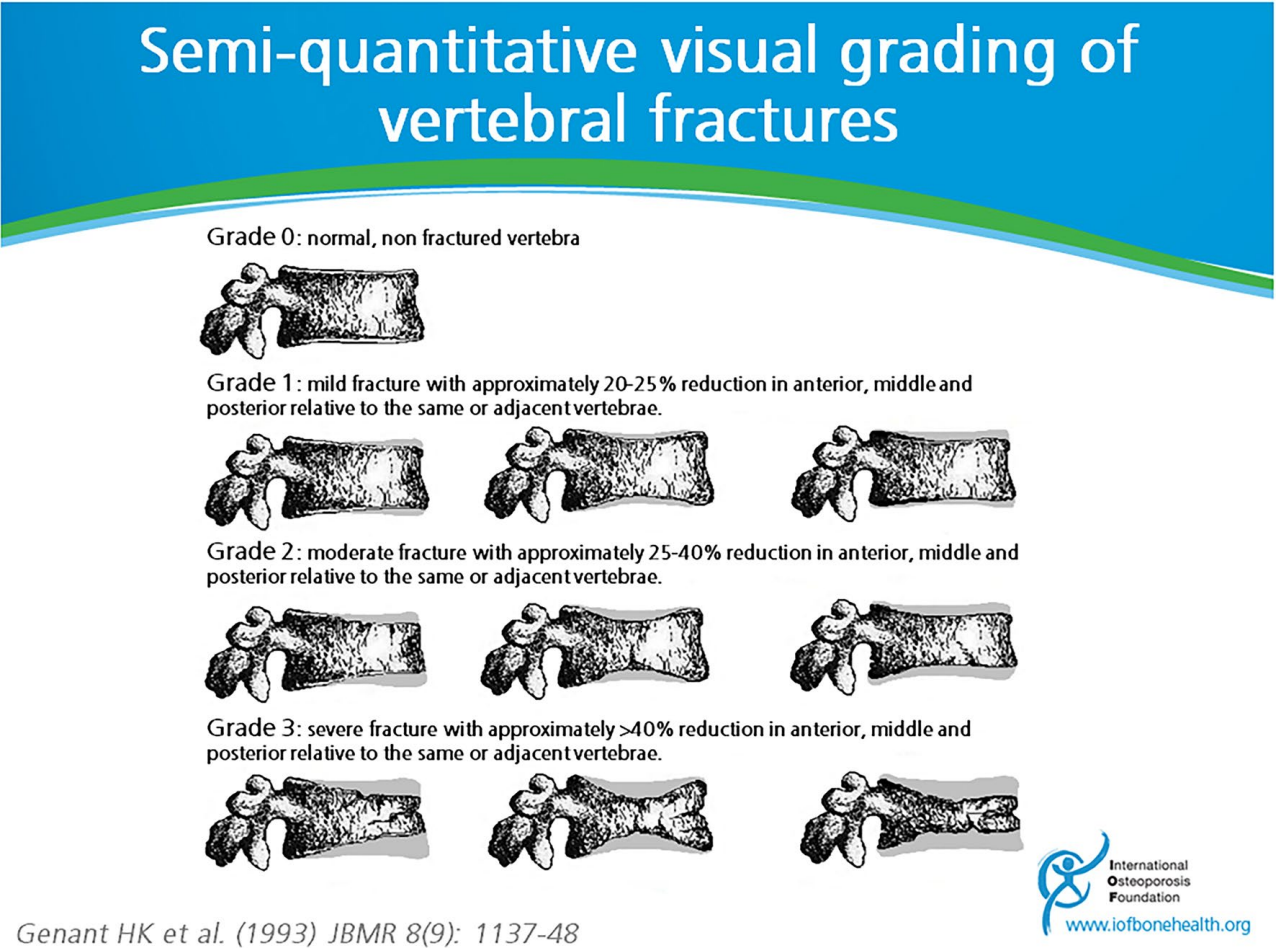
Genant’s semi-quantitative vertebral fracture classification

The basic data of all the subjects were collected, including age (years), height (cm), weight (kg), PSA level (ng/mL), Gleason grade, treatment time (years), and treatment status (with or without ADT). Digital radiography was used to acquire thoracic radiographs. According to Genant’s semi-quantitative method, as shown in Fig. 1, the T4–L1 vertebrae of lateral thoracic radiographs of all subjects were evaluated. Evaluation criteria: According to the degree of vertebral height change (Hx), they were divided into four grades: ① Normal (Grade 0): Hx < 20%; ② Mild fracture (Grade 1): 20% ≤ Hx ≤ 25%; ③ Moderate fracture (Grade 2): 25% < Hx ≤ 40%; ④ Severe fracture (Grade 3): Hx > 40% (Fig. 1).

### Statistical analysis

SPSS 20.0 statistical software was used to process and analyze the data. Continuous measures were expressed as mean with standard deviation (SD), median and range, while dichotomous variables were expressed as numerical values and percentages. Pearson *χ*^2^ test was used to compare the difference between groups, and the *χ*^2^ segmentation method was used to compare the data rate between multiple groups. Data of continuous variables (age and BMI) were compared by univariate analysis or the Kruskal–Wallis test and the Bonferroni post-hoc. The chi-square test was used to compare the differences in the age groups in terms of PSA level, Gleason grade, treatment time, and treatment status (with or without ADT). Multivariate logistic regression analysis was used to analyze the indicators with statistically significant differences. Statistical significance was set at 0.05. The sample size required for the study was estimated by PASS software, and all the data fit the normal distribution.

## Results

### Chi-square test findings

The chi-square test was performed between the control group and prostate cancer patients before treatment (pre-treatment group). The results showed that the vertebral fracture rate in the control group was significantly higher than that in the pre-treatment group (*P* = *0.028*; Table 2). The control group was then compared with the prostate cancer patients after treatment (post-treatment group). The results showed no statistical significance in the vertebral fracture rate between the control and the post-treatment groups (*P* = *0.758*; Table 3). The vertebral fracture rate and moderate-to-severe vertebral fracture rate of pre- and post-treatment groups were also compared. Our results showed that the vertebral fracture rate in the post-treatment group was significantly higher than that in the pre-treatment group (*P* = *0.012*); however, the moderate-to-severe vertebral fracture rate was not significantly different between the two groups (*P* = 0.108), as shown in Tables 4 and 5.

**Table 2:** Comparison of vertebral fracture rates between the control group and the pre-treatment group

Group	Without vertebral fracture	With vertebral fracture	Vertebral fracture rate
Control group	29	71	29%
Prostate cancer patients before treatment	16	84	16%
*P* = 0.028 (< 0.05)

**Table 3:** Comparison of vertebral fracture rate between the control group and the post-treatment group

Group	Without vertebral fracture	With vertebral fracture	Vertebral fracture rate
Control group	29	71	29%
Prostate cancer patients after treatment	31	69	31%
*P* = 0.758 (> 0.05)

**Table 4:** Comparison of the incidence of vertebral fracture in pre- and post-treatment groups

Group	Without vertebral fracture	With vertebral fracture	Vertebral fracture rate
Prostate cancer patients before treatment	16	84	16%
Prostate cancer patients after treatment	31	69	31%
*P* = 0.012(< 0.05)

**Table 5:** Comparison of the incidence of moderate and severe vertebral fractures in pre- and post-treatment groups

Group	Without moderate to severe vertebral fracture	With moderate to severe vertebral fracture	Moderate to severe vertebral fracture
Prostate cancer patients before treatment	85	15	15%
Prostate cancer patients after treatment	76	24	24%
*P* = 0.108(> 0.05)			

### Univariate analysis findings

According to Genant’s semi-quantitative classification of vertebral fracture, prostate cancer patients after treatment were divided into group A (with normal vertebrae), group B (with mild vertebral fracture), and group C (with moderate to severe vertebral fracture). Univariate analysis was performed on the age, BMI, PSA level, Gleason grade, treatment time, and treatment status of the post-treatment group. Significant differences were observed in the age, PSA levels, and treatment parameters among groups A, B, and C (*P* = 0.014, 0.007, 0.043). Significant differences were observed in age and PSA level of groups A and B (*P* < 0.05). The treatment parameters were significantly different between groups A and C (*P* < 0.05). PSA levels were significantly different between groups B and C (*P* < 0.05), as shown in Table 6.

**Table 6:** Univariate analysis of factors affecting vertebral fracture

	Group A (n = 69): Grade 0	Group B (n = 7): Grade 1	Group C (n = 24): (Grade 2, 3)	*P*
Age	68.29 ± 8.05^#^	76.43 ± 7.03^*^	71.71 ± 6.97	0.014
BMI (kg/cm^2^)	23.31 ± 3.03	22.02 ± 2.77	22.15 ± 3.25	0.214
PSA (ng/mL)				
≤ 4	0 (0)^#^	2 (100)^*,△^	0 (0)^#^	0.007
(4–10)	15 (68.2)	0 (0)	7 (31.8)	
(10–20)	14 (93.3)	0 (0)	1 (6.7)	
> 20	40 (65.6)	5 (8.2)	16 (26.2)	
Gleason grade				
≤ 7	32 (60.4)	5 (9.4)	16 (30.2)	0.137
> 7	37 (78.7)	2 (4.3)	8 (17.0)	
Treatment time (years)				
≤ 2	50 (69.4)	3 (4.2)	19 (26.4)	0.168
> 2	19 (67.9)	4 (14.3)	5 (17.9)	
ADT				
Yes	66^△^	7	19^*^	0.043
No	3	0	5	

The three groups were compared in pairs; ^*^ Compared with group A, the difference was statistically significant (*P* < 0.05); ^#^ Compared with group B, the difference was statistically significant (*P* < 0.05); ^△^ Compared with group C, the difference was statistically significant (*P* < 0.05)

*BMI* body mass index, *PSA* prostate-specific antigen, *ADT* androgen deprivation therapy

### Multivariate analysis findings

Patient age, PSA levels, and treatment parameters were introduced into the logistic regression model. Age was found to be a risk factor for vertebral fracture. With an increase in age, the risk of vertebral fracture increases, and the probability of vertebral fracture is 1.076 times than that of one year ago. PSA levels and treatment parameters were not found to be statistically significant in the multivariate analysis, as shown in Table 7.

**Table 7:** Multivariate analysis of factors affecting the vertebral fracture

Factor	B	S.E,	Wald	Sig	Exp (B)	EXP(B) 95% C.I	
						lower limit	Upper limit
Age	0.073	0.031	5.633	0.018	1.076	1.013	1.144
PSA	0.066	0.256	0.067	0.795	1.069	0.647	1.764
ADT	1.345	0.793	2.877	0.090	3.837	0.811	18.147
Constant	− 6.331	2.447	6.696	0.010	0.002		

B: regression coefficient, S.E: standard error, Wald: chi-square value

*PSA* prostate-specific antigen, *ADT* androgen deprivation therapy

### Correlation coefficient test

**Fig. 2:**
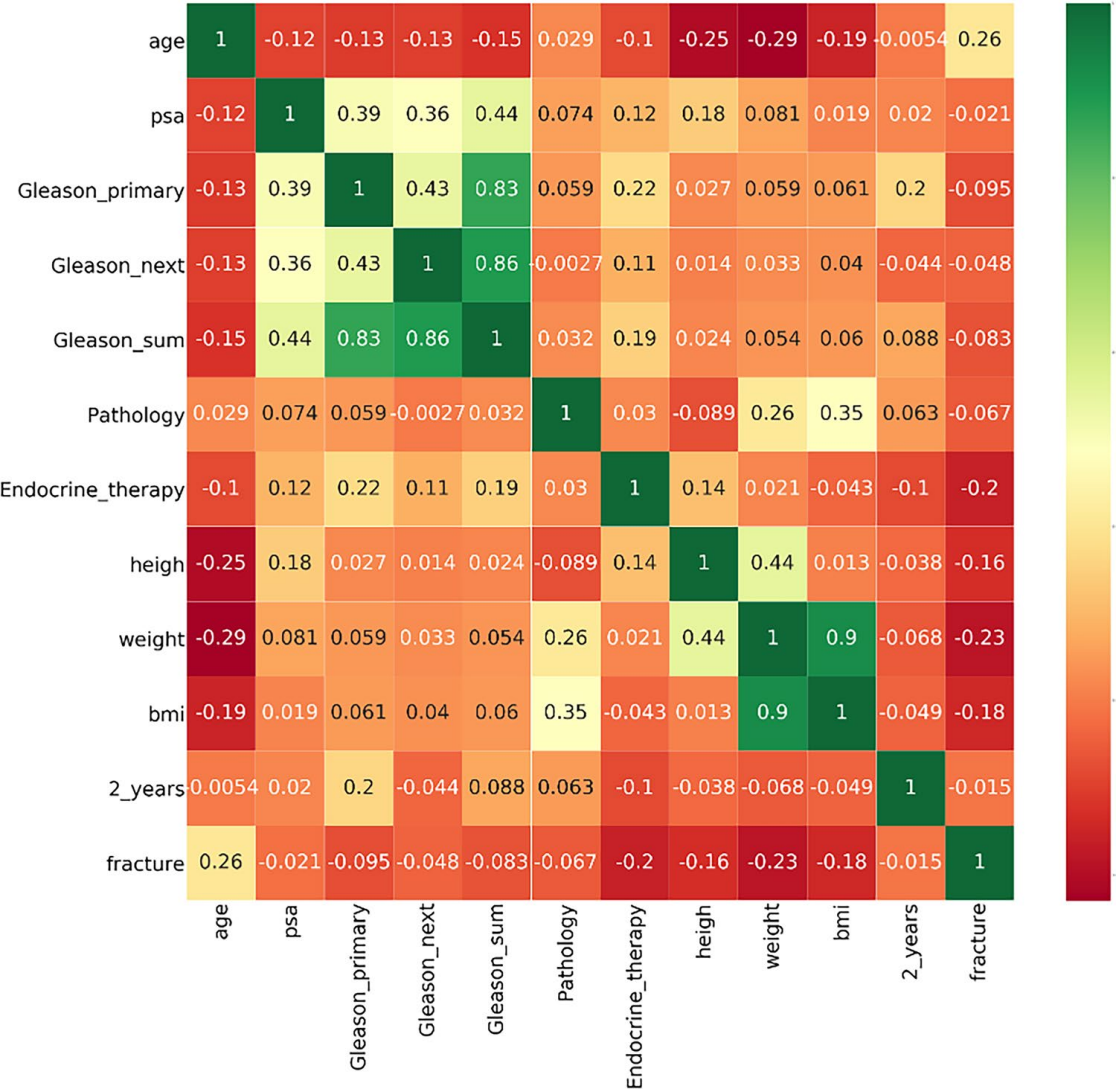
Correlation coefficient helps check the direction and degree of the variation trend between two variables. Values range from − 1 to + 1. The value of 0 indicates that the two variables are not correlated. Positive values indicate a positive correlation, and negative values indicate a negative correlation. PSA prostate-specific antigen, BMI prostate-specific antigen

As shown in Fig. 2, the endocrine therapy positively correlated with the main structure of Gleason grade, with a correlation coefficient of 0.22.Weight and height were negatively correlated with age, and the correlation coefficients of − 0.29 and − 0.25, respectively. Body weight and BMI were positively correlated with pathological types of prostate cancer, with correlation coefficients of 0.26 and 0.35, respectively. The duration of treatment positively correlated with the main structure of Gleason grade. The correlation coefficient between fracture and age was 0.26, while fracture was negatively correlated with endocrine therapy, weight, and height.

## Discussion

In this study, the vertebral fracture rate of the pre-treatment group was found to be significantly lower than that of the control group. This finding might be attributed to the high androgen level of prostate cancer patients. There are abundant levels of androgen receptor (AR), estrogen receptor α (ERα), and estrogen receptor β (ERβ) on the surface of osteoblasts, osteoblasts, and osteoclasts in the human body. Androgen and estrogen play a regulatory role in bone formation and absorption by acting on the abovementioned receptors. Androgens impact the function of osteoblasts through receptors, including the proliferation, synthesis, and secretion of various growth factors and cytokines of osteoblasts, and the production of bone matrix proteins (collagen, osteocalcin, and osteoblast), which play a role in regulating and balancing bone metabolism. Both testosterone and dihydrotestosterone can suppress bone resorption by inhibiting bone resorption stimulators, such as parathyroid hormone (PTH), interleukin 1 (IL1), and interleukin 6 (IL6). In addition, testosterone can promote the secretion of calcitonin, suppressing the activity of osteoclasts. The decrease in calcitonin levels post-castration may also be responsible for the increased activity of osteoclasts. Osteoporosis is a kind of bone disease wherein the patient is prone to fractures due to a decrease in bone mass and the destruction of the fine structure of bone tissue. In prostate cancer patients, androgen promotes the growth of prostate cancer cells. Both surgery-based and drug-based castrations reduce the level of androgen in the body. Although androgen reduction can prolong the life span of patients and reduce the risk of advanced prostate cancer, it also leads to the loss of the regulating effect of androgen on bone formation and bone resorption, eventually causing osteoporotic fractures. The results of this study corroborated this phenomenon. The vertebral fracture rate of the post-treatment group was significantly higher than that in the pre-treatment group. In our previous research on osteoporotic fracture-related factors, we found that treatment time was a risk factor for vertebral fractures in breast cancer patients, and the risk of vertebral fractures in patients being treated for > 2 years increases by 2.736 times. However, there was no significant difference in the vertebral fracture rates between the post-treatment group and the control group, as well as between the prostate cancer patients being treated for > 2 years and those treated for < 2 years. This finding may be attributed to the short duration of ADT treatment for most patients in the case group. In a large study on long-term ADT therapy, the prevalence of osteoporosis was found to be 35% at the beginning, which increased to 43% after 2 years and 81% after 10 years of ADT [8]. Further, in the post-treatment group, univariate analysis showed that age, PSA levels, and treatment parameters significantly correlated with the vertebral fracture rate. However, in the multivariate analysis, only age was found to be significantly associated with vertebral fracture rate. This finding might be attributed to the small sample size or because there is certain collinearity between the selected independent variables.

Prostate cancer patients are prone to bone metastasis, which can cause different degrees of dysfunction, resulting in bone pain, bone fractures, pathological fractures, and other complications. The mechanism underlying bone metastasis development in prostate cancer patients is still unclear; however, it is suspected to be related to direct tumor expansion, retrograde venous flow, and tumor embolism. The most common sites of bone metastasis in prostate cancer patients are pelvis, spine, and ribs. Bone metastasis to the spine can also cause vertebral compression fractures. Lateral radiographs of the thoracolumbar spine can be used to determine whether the vertebral fracture is osteogenic and, thus, can help rule out osteoporotic or metastatic fractures. Clinically, 99 mTc-MDP bone imaging is one of the important modalities used for the diagnosis of bone metastases in prostate cancer patients. However, because prostate cancer patients are older and their own lesions, such as osteoporosis and degeneration, can lead to a positive bone scan and reduced specificity, it is often necessary to combine other imaging methods for bone scan diagnosis. Magnetic resonance imaging (MRI) also plays an important role in the detection of bone metastases in prostate cancer patients. In particular, 3D whole-body MRT1-weighted sequential imaging has better diagnostic performance than planar MRI for bone metastases. Currently, the gold standard for the diagnosis of osteoporosis is still the determination of BMD using dual-energy X-ray absorptiometry (DXA). In previous studies, we found that BMD measurement, combined with vertebral fracture assessment (VFA), in postmenopausal women can help detect occult vertebral fractures and improve the clinical diagnosis of osteoporosis [9]. A recent epidemiological survey also showed that, according to the DXA bone density test results, the prevalence rate of osteoporosis in Chinese men over 50 years old was 6.46% [10]. In addition, osteoporotic fractures occur in one out of five men over 50 years old. Also, the consequences of osteoporotic fractures are more serious in men, with significantly higher disability and fatality rates than in women [11, 12]. However, at present, DXA is not popular, and clinicians do not pay much attention to it. Another survey found that the awareness rate of osteoporosis in men over 20 years old was only 10.5%, while 25% of the men were subjected to the BMD test. Only 7% of people over 50 years old were aware of osteoporosis, and only 3.2% of men had their bone density tested. Of the 100 prostate cancer patients, only 25% were ever subjected to a DXA test. Of those 25 patients, 56% had osteoporosis, 28% had low bone mass, and only 16 percent had normal bone density. Genant’s semi-quantitative assessment is the basis for standardized interpretation of the severity of vertebral fractures. It is a simple and effective method for detecting vertebral fractures. Early detection of vertebral fractures can help improve the diagnosis and prognosis of osteoporosis [7]. In the 2020 guidelines of the American Association of Clinical Endocrinologists (AACE), osteoporosis is described as “a silent bone disease” that is preventable and treatable. Lumbar (thoracic) lateral vertebral imaging or DXA VFA evaluation is recommended when the T value of BMD is < − 1.0, and at least one of the following conditions exists: ① Age: ≥ 70 years old female or ≥ 80 years old male; ② Height: Previous reduction > 4 cm; ③ History of vertebral fracture: Self-reported but not treated; ④ Glucocorticoid: ≥ 5 mg prednisone daily for ≥ 3 months [13]. Prostate cancer patients treated with ADT are at risk for osteoporotic fractures. Lateral chest radiography is a routine examination method for inpatients. Radiologists can use lateral chest radiographs to preliminarily assess a vertebral condition, thus prompting clinicians to pay attention to the bone health of patients receiving ADT treatment and for further diagnosis and treatment.

This study also has some limitations. First, the evaluation of vertebral fractures was based on Genant’s semi-quantitative method. Although it is currently a commonly used diagnostic method, the influence of the evaluator’s subjectivity cannot be excluded. Second, this study did not include other osteoporosis-related factors, such as smoking, drinking, obesity, and lack of exercise. Third, the small sample size might lead to statistical bias. Although this study has some deficiencies, it also provides a reference value for future research directions.

## Conclusions

The incidence of osteoporotic vertebral fracture in prostate cancer patients before treatment was lower than that in the normal population. However, this incidence increases significantly after treatment, primarily due to age, PSA level, and treatment parameters. Osteoporotic fractures are an important complication of ADT. To prevent these fractures, all patients subjected to ADT should be closely monitored for bone health by lumbar (thoracic) lateral imaging or VFA with DXA. Their monitoring might help reduce the incidence of fractures through lifestyle and necessary pharmacological interventions, thereby improving the quality of life of the patients.

## Data Availability

The data and supportive information is available within the article.
